# The Role of Monocytes and Macrophages in Autoimmune Diseases: A Comprehensive Review

**DOI:** 10.3389/fimmu.2019.01140

**Published:** 2019-05-24

**Authors:** Wen-Tao Ma, Fei Gao, Kui Gu, De-Kun Chen

**Affiliations:** ^1^Veterinary Immunology Laboratory, College of Veterinary Medicine, Northwest A&F University, Yangling, China; ^2^School of Life Sciences, University of Science and Technology of China, Hefei, China

**Keywords:** monocyte, macrophage, autoimmune disease, count, function, polarization

## Abstract

Monocytes (Mo) and macrophages (Mϕ) are key components of the innate immune system and are involved in regulation of the initiation, development, and resolution of many inflammatory disorders. In addition, these cells also play important immunoregulatory and tissue-repairing roles to decrease immune reactions and promote tissue regeneration. Several lines of evidence have suggested a causal link between the presence or activation of these cells and the development of autoimmune diseases. In addition, Mo or Mϕ infiltration in diseased tissues is a hallmark of several autoimmune diseases. However, the detailed contributions of these cells, whether they actually initiate disease or perpetuate disease progression, and whether their phenotype and functional alteration are merely epiphenomena are still unclear in many autoimmune diseases. Additionally, little is known about their heterogeneous populations in different autoimmune diseases. Elucidating the relevance of Mo and Mϕ in autoimmune diseases and the associated mechanisms could lead to the identification of more effective therapeutic strategies in the future.

## Introduction

Monocytes (Mo) and macrophages (Mϕ) possess broad immuno-modulatory, inflammatory, and tissue-repairing capabilities and actively participate in the development of many autoimmune diseases (1). These cells can secrete a wide range of cytokines and chemokines, which stimulate and recruit additional immune cells to diseased tissue (2). In many autoimmune diseases, the presence of autoantibodies and autoreactive B and T cells indicates that adaptive immune system is critical for pathogenesis, but this cannot fully account for the development of autoimmune diseases, and the innate immune response may play a necessary and irreplaceable role as well (1, 3). In fact, Mo or Mϕ infiltration is generally observed in many autoimmune diseases (4–13). Additionally, a change in the count or frequency of Mo/Mϕ is a hallmark of several autoimmune diseases, i.e., systemic sclerosis (SSc), rheumatoid arthritis (RA), primary biliary cholangitis (PBC), Sjögren's syndrome (SS), and inflammatory bowel disease (IBD) (4, 5, 10, 14–17). However, it should be noted that Mo/Mϕ frequency and count in the peripheral blood or afflicted tissues can be affected by several factors including at least bleeding regimes (for instance time of bleeding) and status of the patients (medical treatment, food intake, age, sex etc.). Thus, Mo/Mϕ frequency and count and their correlation with disease stage are usually controversial in different studies.

Although the regulatory mechanism of Mo and Mϕ in the development of autoimmune diseases has not been fully elucidated, consensus appears to suggest that their abnormal activation plays a key role. Typically, M1-polarized Mϕ are pro-inflammatory and secrete interleukin (IL)-12 and tumor necrosis factor (TNF)-α to contribute to local inflammation, while M2-polarized Mϕ produce IL-4 and IL-10 that mount immunomodulatory, wound repair and tissue remodeling functions [as reviewed by Funes et al. (18)]. However, the M1/M2 dichotomy may oversimplify a more complex activation mechanism. In fact, in certain autoimmune diseases, both M1- and M2-polarized Mϕ are detected simultaneously, and both M1- and M2-stimulating cytokines are present on a large scale (19–22). Additionally, Mϕ even exhibit an intermediate activation status by co-expressing both M1- and M2-specific markers in certain diseases (23, 24). Furthermore, in many cases, Mϕ polarization is a dynamic and reversible event that depends upon the local environment and stage of disease (25).

In the present review, we will discuss our current understanding of the properties of Mo/Mϕ in certain autoimmune diseases, highlighting the phenotypical, functional, and activation properties of these cells in disease pathogenesis and the relevant mechanisms (Summarized in Tables 1, 2). Because there are very limited reports regarding the role of Mo/Mϕ in autoimmune Addison's disease, autoimmune thyroid disease, antiphospholipid syndrome, and myasthenia gravis, these four diseases are not discussed in the present article.

**Table 1:** Characteristics of Mo and Mϕ in autoimmune diseases.

**Disease**	**Percentage/count alterations**	**Functional abnormalities**	**Polarization profiles**
SLE	Similar to healthy controls in Mϕ number (26); Decreased Mϕ count (27). Increased CD14^+^CD16^+^ Mo number (26).	Increased expression levels of CD40 (28), CD86 (29, 30), ICAM-1 (31, 32), Siglec-1 (33); Defective phagocytic ability (34–36).	M1 polarization: Higher levels of IL-1β (37), IFN-γ (19), CXCL10 (38), CCL2 (39), GM-CSF (40). M2 polarization: Higher levels of IL-10 (20, 21).
SSc	Number: Increased CD68^+^ Mϕ (41); Increased CD14^+^ Mo (42); Increased CD16^+^ Mo in diffuse SSc (42). Percentage: Increased CD14^+^ Mo (43).	More profibrotic (44); Increased expression of Siglec-1 (45).	M2 polarization: Higher levels of IL-4, IL-10, IL-13, TGF-β, and PDGF (46–48). Increased expression of CD163 and CD204 (41, 43).
RA	Increased number and percentage of Mϕ (4, 5).	Increased Mo CD80 (49), CD276 (49), and Siglec-1 expression (50).	M1 polarization: Higher levels of TNF-α, IL-1, IL-6, and IL-12 (51–55). Increased expression of CD50 and CD36 while lower expression of CD163 and CD209 (56). Higher M1/M2 Mo ratio (57).
MS	Increased total mononuclear phagocyte number (11, 12, 58, 59).	Increased expression of CD68, HLA and CD86 (60). Abnormal metabolic changes (more glycolysis) (61).	An intermediate status: Co-expression of CD40 and mannose receptor (24).
T1D	Increased CD14^+^ Mo number (62). Decreased CD16^+^ Mo number (62).	Decreased phagocytosis ability (63, 64). Cytolytic to islet β-cells (65).	M1 polarization: Higher levels of C-reactive protein (66), IFN-γ (67), CXCL10 (68), CCL2 (68), IL-6 (66, 69), IL-1β (66, 69), TNF-α (70, 71).
PBC	Increased Kupffer cell number in stage 3 and 4 cases (10, 72). Similar number of Kupffer cells at different stages (73). Increased liver CD14^+^ Mo number (73). Increased circulating CD14^high^CD16^+^ and CD14^low^CD16^+^ Mo number (74).	More sensitive to TLR ligation (75). Increased Siglec-1 expression (76). Recognition of AMA-apotope complexes (77).	M1 polarization: Higher levels of IL-1β, IL-6, IL-8, IL-12, and TNF-α (75, 78). Increased endotoxin production of biliary epithelial cells (79). Increased expression of CD40L (72).
SS	Increased CD14^high^CD16^+^ and CD14^low^CD16^+^ Mo number (15, 80).	Decreased phagocytosis ability (81).	M1 polarization Increased levels of IL-6 (82), IL-12 (83), IFN-γ (84), TNF-α, IL-1β, IL-18, CXCL8, and CXCL10 (80, 85–87). Activation of Mϕ NFκB signaling pathway (88).
Celiac disease	Increased CD68^+^ Mϕ number (7).	Decreased phagocytosis ability (7, 89). Increased antigen-presenting ability (90, 91).	M1 polarization Higher levels of IFN-γ, IL-1β, TNF-α, and IL-8 (22, 90). Increased expression of CD80, CD86, and CD40 (88). Activation of NFκB signaling pathway (88). M2 polarization: Higher levels of IL-4 and IL-10 (22). Increased expression of arginase 1 and 2 after stimulation (92, 93).
IBD	Increased CD68^+^ Mϕ number in UC and CD (8, 9, 16). Increased CD163^+^ Mϕ number in CD (16). Increased circulating CD14^+^CD16^+^ while decreased CD14^hi^CD16^−^ Mo in CD (94, 95).	Decreased retinoic acid synthesis ability in CD (8). Abnormally accelerated lysosomal degradation of cytokines in CD (96). Defective GM-CSF receptor expression and function in UC and CD (97).	M1 polarization: Increased production of IL-23 and TNF-α in UC (98, 99). Suppressed IL-10 production in UC (98, 99). Higher expression of CD16/32 in UC (98, 99). M2 polarization: Higher IL-13 level in CD (100). Higher CD163 expression in CD (16). Higher CD163 and CD206 expression in UC (16, 101).

**Table 2:** Mechanisms of Mo/Mϕ activities in autoimmune diseases.

**Diseases**	**Triggers for Mo/Mϕ recruitment and activation**	**Molecular mechanisms of Mo/Mϕ function**	**Mo/Mϕ-derived mediators in disease progression**
SLE	TNF-α: Mo NF-κB inflammatory response (102). Anti-dsDNA antibodies: NLRP3 inflammasome activation in Mϕ (103). Microparticle-associated immune complexes: activation of pro-inflammatory Mo (104). IFN-α: B-lymphocyte stimulator expression in Mo (105). Anti-C1q autoantibodies: induction of a pro-inflammatory phenotype in Mϕ (106). HMGB1: Mϕ inflammatory responses (107).	Decreased PPAR-γ, KLF2 and KLF4 expressions: Defective phagocytosis (108, 109). Decreased PPAR-γ expression: pro-inflammatory functions (110). Increased IRF1 expression: enhanced inflammasome activity (111).	IL-1β, IL-6, TNF-α and IL-10: mediating local and systemic inflammation (112–115).
SSc	CCL2: Mo/Mϕ recruitment (116). Type I IFN: Mϕ activation (45). PDGF-BB: dermal infiltration of Mo/Mϕ (117). CX3CL1: Mo/Mϕ recruitment (118). MIF: concentrating Mϕ at inflammatory loci (119).	TLR/MyD88 signaling and the transcription factor Fos-related antigen 2: TIMP1 production by Mo (120, 121).	PDGF and TGF-β: fibrosis development (44, 117, 122) CCL4, CXCL8, and CXCL10: tissue inflammation and fibrosis (123). CXCL13: fibrosis development (124). Versican and CCL2: Mo recruitment (125). TIMP-1: fibrosis development (121, 126).
RA	CCL2: Mo recruitment (13). Activin A: generation of pro-inflammatory Mϕ (56). Neutrophil microvesicles: preventing inflammatory activation of Mϕ (127). GM-CSF and osteopontin: Mo migration (128). MicroRNA-155: survival of Mo (129, 130).	NFAT5: survival of activated Mϕ (131). Succinate/GPR91 signaling: IL-1β production from Mϕ (132). Liver X receptor pathway: potentiating TLR-driven cytokine production from Mϕ (133).	IL-1, IL-6, IL-12, and TNF-α: mediating local and systemic inflammation (134, 135). IL-1, IL-6, and TNF-α: mediating cartilage degradation (136).
MS	CCL2: M1 macrophage recruitment (137). GM-CSF: migration of Mo across the blood brain barrier (138, 139). IFN-γ and α-B-crystallin: activation of microglia/Mϕ of MS-affected brain tissue (140). Acetylcholine-producing NK cells: kill and inactivate CCR2^+^Ly6C^hi^ Mo (141).	Decreased SHP1 signaling: enhanced inflammatory activity of Mo (142). KLF2: negatively regulate Mϕ activation (143).	NLPR3 inflammasome: T cell recruitment (144). IL-1β, IL-6, and IL-23: Th17 cell generation (145–147). TNF-α, IL-6, IL-12, IL-1β, Reactive oxygen, and nitrogen species: mediating inflammatory responses (140, 148, 149). IL-6 and BAFF: B cell survival and differentiation (150).
T1D	CCL2: Mϕ recruitment (151). MIF: activating Mϕ and driving Th1 cell response (152–154). GM-CSF: Mo activation (155). Acetoacetate: IL-6 and ROS production from Mo (156) and Mo adhesion to endothelial cells (157). Myeloid-related proteins: adhesion of Mo to fibronectin (158).	Increased expression of long-chain acyl-CoA synthetase 1: enhanced inflammatory activity (159). Increased LFA-1 expression: Adhesion to endothelial cells (157). Persistent activation of STAT5: aberrant inflammatory gene expression (155).	IL-1 and IL-6: Th17 cell generation (69).
PBC	CX3CL1: Mo recruitment (160). MIF-3α, osteopontin and CCL2: MDM recruitment (161–163). TLR ligands: Mϕ activation and production of pro-inflammatory cytokines (75, 78). AMA-apotope complexes: MDM activation (164). TNF-α-induced protein 8-like-2: productions of TNF-α, IL-1β, and IL-8 by Mo (165). Exosomes: expression of co-stimulatory molecules on Mo (166).	TNF-α-induced protein 8-like-2 signaling: inhibiting Mo NF-κB pathways and Mo activation (165).	IL-12: differentiation of Th1 cells (74). NLPR3 inflammasome: inducing IL-1β production and promoting differentiation of Th17 cells (167). IL-1β, IL-6, IL-8, IL-12, and TNF-α: promoting liver inflammation and injury (75, 78)
SS	CXCL9 and CXCL10: migration of CXCR3^+^ Mϕ (168). MIF: local infiltration of Mϕ (119). Extranuclear accumulation of DNA: NLRP3 inflammasome activation (169).	MicroRNAs: targeting the canonical TGF-β signaling pathway as opposed to pro-inflammatory IL-12 and TLR/NF-κB pathways (170). Activated NF-κB pathway: amplifying cytokine production and inflammatory response (88).	CCL22: enhancing autoreactive T cell response and recruitment (171). IL-6, IL-18, type I IFN, and BAFF,: mediating pro-inflammatory immune responses (87, 172, 173)
Celiac disease	Gliadin peptides: Mo production of pro-inflammatory cytokines and chemokines (90, 90, 174) IL-15: supporting Th17 and Th1 responses (175).	TLR/MyD88 signaling pathway: mediating pro-inflammatory cytokine production (176–178). NF-κB activation: Mo production of IL-8 and TNF-α (174). TLR4/MyD88/TRIF/MAPK/NF-κB signaling pathway: production of IL-1β by Mϕ (179) lncRNA: facilitating Mϕ inflammatory gene expression (180). Increased STAT3 signaling: Mo activation and IFN-γ production (181).	Tissue transglutaminase: involved in processes contributing to inflammation (182). IL-1β, IL-23, TNF-α, IL-6, IFN-γ: tissue inflammation (179, 181, 183, 184).
IBD	IL-33: induction of Mϕ with tissue-repairing ability (185). Luminal extracellular vesicles: Mϕ migration (186). Gut microbiota (Clostridium butyricum): induction of IL-10-producing Mϕ (187).	PPAR-γ mutation: generation of pro-inflammatory M1 Mϕ (188, 189). Higher expression of Nuclear paraspeckle assembly transcript 1: mediation of the inflammatory responses through exosome-mediated polarization of Mϕ (190).	IL-1β, IL-6, IL-23, TNF-α and TNF-like protein 1A: generation of Th1 and Th17 cells (191–194). IL-23: promoting Th17 cell differentiation and NK cell activation (195–197). NLRP3 inflammasome: promoting experimental IBD development (without detailed mechanisms) (198).

## Mo and Mϕ in Autoimmune Diseases

### Systemic Lupus Erythematosus (SLE)

Mo percentage and count have been analyzed in SLE patients, but the findings vary among different studies. One group found that although the absolute number of the whole Mo population was similar between SLE patients and healthy controls, the rate and absolute number of CD14^+^CD16^+^ Mo was significantly higher in SLE patients, and steroid therapy could down-regulate the percentage and number of these cells in a dose-dependent manner (26). In contrast, a more recent study based on 205 SLE patients and 74 healthy controls reported decreased absolute Mo counts in SLE patients (27). However, there was no significant difference in the proportions of various Mo subpopulations. In addition, neither the absolute count nor the percentage of various Mo subsets was associated with disease activity (27). It appears that the reduction in Mo count in the latter study is supported by an independent study, which showed that Mo and Mϕ are more fragile and likely to undergo apoptosis (analyzed by flow cytometry with annexin V and propidium iodide) when induced by the sera of SLE patients (199). Although the detailed mechanism remains unclear, it appeared that C5a complement was involved in this process while serum IgG autoantibody was not involved, since Mo apoptosis profile correlated positively with C5a level, and depletion of IgG did not affect such apoptosis. In lupus mice, Mϕ depletion leads to attenuated skin and kidney disease severity, suggesting that these cells do play a critical role in SLE pathogenesis (200).

One of the contributions of Mo/Mϕ to SLE pathogenesis is modulation of the adaptive immune system. The binding of co-stimulatory molecule CD40 to its ligand CD40L is required for the activation of humoral immune responses including B cell activation, plasma cell differentiation, antibody secretion, and isotype-switching (201). In patients with SLE, a significant increase in the frequency of CD40L-expressing peripheral Mo was observed compared with healthy controls (28). Consistent with this finding, although B cells from SLE patients and normal controls showed similar CD40 expression levels, recombinant CD40L significantly stimulated the production of total IgG by SLE B cells but not normal B cells (202). In addition, data from murine studies showed that CD40L overexpression could induce lupus-like autoimmune disease, while CD40L neutralization prevented autoreactive B cell activation and autoantibody production in lupus-prone mice (203, 204). Thus, although direct evidence is still lacking, these data do suggest a potential contribution of Mo to the observed B cell hyperactivity in SLE patients through the CD40/CD40L signaling pathways. Moreover, Mo from SLE patients tend to differentiate into dendritic cells that express higher levels of CD86 when induced by IFN-α in the serum, and this potentiates them with higher abilities to present autoantigens to autoreactive T cells and B cells (29, 30).

Overexpression of adhesion molecules may lead to aberrant Mϕ migration and activation. Mϕ from active SLE patients overexpress intercellular adhesion molecule (ICAM)-1, which is associated with tissue recruitment and inflammatory cytokine production, and this is partially offset by corticosteroid therapy (31, 32). SLE Mϕ also express increased levels of sialic acid–binding Ig-like lectin 1 (Siglec-1, CD169), which could be dramatically reduced by high-dose glucocorticoid treatment (33). However, in view of the broadly anti-inflammatory effect of glucocorticoid (205), it should be noted here that this study could not rule out the possibility that the reduction in Siglec-1 expression level might result from a bystander effect of glucocorticoid treatment. Increased Mϕ Siglec-1 expression may constitute a potent inflammatory signal to promote the activation of autoimmune CD4^+^ or CD8^+^ T cells (206). In fact, it was suggested that Siglec-1 expression in Mϕ could serve as a potential biomarker for lupus activity, as the percentage of Siglec-1-expressing Mϕ was shown to positively correlate with SLE Disease Activity Index and autoantibody levels (33).

Defective phagocytosis of Mϕ has also been suggested to contribute to autoimmunity in SLE. The phagocytic capacity of Mϕ is crucial for the clearance of dead cells and debris, which otherwise can be important sources of autoantigens. Accumulating data from *in vitro* studies and murine models illustrate that ineffective clearance of apoptotic cells by Mϕ might be an important trigger of the autoimmune process in SLE. Two decades ago, it was observed that non-inflammatory phagocytosis of apoptotic cells by Mo-derived Mϕ (MDMs) was strikingly impaired in SLE patients (34). In addition, apoptotic cells were found to accumulate in the germinal centers of the lymph nodes in patients with SLE, and *in vitro* uptake of autologous apoptotic cells into Mo-derived Mϕ from SLE patients was significantly impaired (35). Interestingly, the percentage of apoptotic polymorphonuclear neutrophils (PMN) from SLE patients was significantly higher than that from healthy controls, and this percentage correlated positively with SLE Disease Activity Index and serum levels of autoantibodies (207). In addition, the phagocytosis defect may be compounded by the serum milieu of SLE patients because serum from these patients had a strong capacity to accelerate the apoptosis rate of PMN and Mϕ, which might further contribute to the high load of potential autoantigens (199, 207). Based on data from murine models, it was found that Mϕ with low expression of TLR9 and decreased TLR responsiveness to nucleic acids were largely responsible for the immunologically silent clearance of apoptotic cells (108), which was consistent with the finding that TLR9 was required in SLE pathogenesis (208). In addition, transcription factors Kruppel-like factors 2 (KLF2) and 4 (KLF4) are also important regulators of apoptotic cell clearance in SLE prone mice (108). Intriguingly, it appears that cues from the tissue microenvironment dictated these characteristics of Mϕ, as removal of these cells from specific tissues resulted in their inability to engulf apoptotic cells without generating inflammatory responses (108). A second feature of the impaired phagocytic capacity of SLE Mϕ is the delayed clearance of immune complexes (IC). Elegant work by Michael M. Frank and coworkers examined the half-time of IgG-sensitized, ^51^Cr-labeled erythrocytes as a measure of Fc receptor function (209). They showed that Fc-specific clearance rates were strikingly prolonged in 13 of 15 patients, and this correlated with elevated levels of IC and with disease activity. Supporting this conclusion, another study by Maria et al. has recently showed that decreased Fc receptor function correlated positively with disease activity and renal involvement (36). In addition, certain Fc receptor polymorphisms appears to determine the clearance of IC *in vivo*, and their heritage is associated with the course of SLE in some ethnic populations (210, 211). Abnormal Mϕ activation has also been observed in SLE patients. Labonte et al. demonstrated that higher activation profiles of Mϕ were associated with more active cases of SLE (212). In addition, Mϕ activation syndrome, a rare but usually very severe or even life-threatening complication has been reported in SLE patients (213, 214).

Accumulating findings suggest the predominance of M1 Mϕ in SLE pathogenesis. Excessive pro-inflammatory M1 Mϕ-related cytokines are produced by Mϕ from SLE patients, including IL-1β (37), interferon (IFN)-γ (19), C-X-C motif chemokine 10 (CXCL10) (38), and C-C motif chemokine ligand 2 (CCL2) (39). In addition, the pro-inflammatory serum milieu of SLE patients also favors M1 polarization, including high levels of IFN-γ, TNF-α, and granulocyte-Mϕ colony-stimulating factor (GM-CSF) (40, 112). M1 dominance may come at the expense of insufficient anti-inflammatory M2 polarization. It was shown that serum transforming growth factor (TGF)-β levels were significantly reduced in lupus patients, and TGF-β levels showed a reverse association with disease activity and organ damage in SLE patients (215). In addition, antibodies against scavenger receptors (an M2 Mϕ marker) or scavenger receptor knockout in lupus prone mice led to a compromised ability of Mϕ to engulf apoptotic cells and resulted in more aggravated SLE symptoms (109).

Considering the pro-inflammatory nature of M1 Mϕ and that M2 Mϕ are anti-inflammatory and are capable of engulfing apoptotic cells during apoptosis, it is reasonable to assume that M1 predominance and M2 insufficiency combine to worsen SLE severity. Indeed, researchers are trying to cure SLE by modulating Mϕ polarization. In a murine model of SLE, adoptive transfer of anti-inflammatory M2a Mϕ induced by IL-4 significantly decreased SLE activity (216). In patients with SLE, pioglitazone treatment enhanced M2 polarization of Mo-derived Mϕ, increasing their anti-inflammatory capacity while suppressing their production of various pro-inflammatory cytokines (217).

Available online at: Intriguingly, large amounts of IL-10, which is a hallmark of M2 Mϕ, are commonly detected in patients with SLE, and serum levels of IL-10 correlate positively with disease activity (20, 21). Contrary to its canonical anti-inflammatory functions, IL-10 in SLE acquires a pro-inflammatory capacity. This is largely dependent on high concentrations of type I IFNs, which confer a pro-inflammatory gain of function upon IL-10 and lead to a positive feedback loop of pro-inflammatory cytokine production (113). Priming of primary human Mϕ with IFN-α resulted in significantly enhanced STAT1 activation in the presence of IL-10, leading to activation of several STAT1-dependent genes such as CXCL9, CXCL10, and IFN regulatory factor 1 (113). In addition, IL-10 can directly stimulate production of platelet-activating factor (a phospholipid mediator of inflammation) of Mo of SLE patients (218). Indeed, IL-10 antagonist administration ameliorated SLE severity effectively during a 6-month therapy, even though this finding was limited by the small sample size of the study (219).

In addition to IL-10, SLE Mo or Mϕ also produce copious IL-6 and TNF-α. Elevated IL-6 levels are positively associated with disease activity or autoantibody levels (114). The underlying mechanism seems to be the stimulation of B cell hyperactivity by IL-6 (220). Indeed, in an open-label phase I dosage-escalation study, IL-6 receptor inhibition showed a significant decrease in the frequency of circulating plasma cells, reduced autoantibody levels in the serum, and significant disease improvement (221). Another cytokine, TNF-α, is generally reported to be elevated in SLE and positively associated with disease activity (112). However, TNF-α blockade therapy in SLE is controversial. Although this therapy was shown to reduce disease severity, autoantibodies to double-stranded DNA and cardiolipin increased during treatment (222). Furthermore, it seems that TNF-α blockade is safe only for short-term treatment, while long-term therapy would likely provoke severe adverse effects such as lymphoma and Legionella pneumonia (223).

Intriguingly, in lupus prone NZB/W and NZW/BXSB mice, nephritic resident CD11b^hi^F4/80^hi^ Mϕ exhibit little arginase- or iNOS-producing ability even after stimulation with M1 or M2 Mϕ-inducing cytokines, irrespective of the clinical status of the mice (224). Instead, these kidney residents show a mixed pro- and anti-inflammatory phenotype during lupus nephritis (224). In contrast, Mo-derived Mϕ of the same mice were readily responsive to cytokine stimulation and can be induced to differentiate into the correspondingly M1 or M2 cells (224). In addition to these phenotypic differences, differences, functional analysis showed that nephritic resident Mϕ had higher antigen-presenting function and phagocytosis ability compared with MDMs of the kidney (224).

Several molecules and pathways have been suggested to be associated with controlling polarization and inflammatory profiles of Mϕ. For example, using MDMs of normal subjects and SLE patients, Saeed et al. found that epigenetic modification is partly responsible for the Mϕ polarization profile in SLE (225). Their data showed that sodium valproate, an histone deacetylase inhibitor, can potently induce the alternative activation of Mo-Mϕ *ex vivo* and inhibit the pro-inflammatory profile of these cells when stimulated by apoptotic cells *in vitro* (225). The same group also found that aryl hydrocarbon receptor (AhR)-mediated signaling pathway is responsible for the secretion of anti-inflammatory cytokines and expression of M2 markers from MDMs of SLE patients, as AhR agonist treatment of these cells led to a significant downregulation of M1 markers and expression of pro-inflammatory cytokines, coincided with an upregulation of M2 markers and expression of anti-inflammatory cytokines (226). In addition, decreased peroxisome proliferator-activated receptor gamma (PPAR-γ) expression has also been proposed to be involved in the defective efferocytosis and abnormal pro-inflammatory characteristics of MDMs from SLE patients (217).

### SSc

More than two decades ago, Ishikawa et al. stained skin specimens from patients with SSc and found that Mϕ infiltration was generally observed around skin adnexa and vessels as well as between collagen bundles, while no close relationship with disease duration was found (6). Later, another group showed that the percentage of peripheral Mo in SSc is significantly higher than that in healthy controls. Notably, this higher percentage of Mo also correlated with worse prognosis and visceral disease involvement (14). However, in this study, Mo were not detected specifically through CD14 but instead were gated indirectly through CD3^+^CD4^−^, weakening the general application of this conclusion. Using a more specific Mϕ marker, another group showed that the number of CD68^+^ Mϕ was significantly higher in the skin of patients with localized SSc (41). The same group also found that the percentage of CD14^+^ circulating Mo was significantly greater in SSc patients than in healthy controls (43). In a more recent study, Lescoat et al. found that SSc patients had an elevated count of total peripheral Mo relative to healthy controls (42). Notably, the CD16^+^ subpopulation count was more significantly increased in diffuse SSc than in limited SSc. In addition, the absolute count of CD16^+^ Mo was significantly associated with the severity of skin fibrosis, pulmonary fibrosis, restrictive ventilatory defect, and pulmonary function impairment, suggesting a potential link between this subpopulation of Mo and the pathogenesis of fibrosis in SSc (42, 227). A potential mechanism underlying the increased Mϕ count may involve Mϕ migration inhibitory factor (MIF), which is capable of suppressing the random migration of Mϕ to concentrate them at inflammatory loci. Selvi et al. has reported the detection of high levels of MIF in the basal and suprabasal keratinocytes of SSc-affected skin (119). In addition, elevated concentrations of MIF in the peripheral blood of patients with diffuse cutaneous SSc were identified.

Several lines of evidence have implicated the functional abnormality of SSc Mo/Mϕ. It was reported that SSc Mo were more pro-fibrotic, as they displayed increased differentiation potential toward type-1 collagen- and α-smooth muscle actin (SMA)-expressing cells after stimulation (44). In addition, the production of tissue-inhibitor of metalloproteinase-1 (TIMP-1), an important protein capable of inhibiting extracellular matrix degradation, is significantly increased in SSc Mo mediated by TLR/MyD88 signaling and the transcription factor Fos-related antigen 2 (20, 21, 126). SSc Mo/Mϕ also show abnormally expressed markers that are associated with certain functions. First, increased expression of Siglec-1 in tissue Mϕ and circulating Mo of SSc patients was identified, suggesting a potential role for type 1 IFN-mediated Mo/Mϕ activation in SSc (45). In line with this finding, levels of IFN-α mRNA were significantly increased in vascular and perivascular cells in skin samples of SSc patients (228). However, how Siglec-1 is induced and to what extent it contributes to the pathogenesis of SSc need further verification. In a recent study, Moreno-Moral et al. explored the contribution of MDMs in mediating genetic susceptibility to SSc. By conducting genome-wide genotyping and RNA-sequencing, their work shows that gene expression in Mϕ from SSc patients is altered, especially higher expression of the *GSDMA* and *GRB10* genes (229). However, the relevance of these results at the protein level has yet to be examined in the future.

Mounting evidence suggests the predominant M2 polarization of Mϕ in SSc. The elegant work of Nobuyo et al. showed an evident increase in the number of CD14^bright^CD163^+^CD204^+^ Mϕ in the fibrotic areas of the SSc skin (41, 43), suggesting that this cell subset may be potential a regulator of fibrosis in SSc skin. Of note, CD204-deficient mice failed to develop silica-induced fibrosis, suggesting a critical role for this scavenger receptor in fibrosis (230). This finding was underpinned by the works of several other groups, which reported that a soluble form of CD163 (sCD163), released from the Mϕ cell surface, was increased in the sera of SSc patients relative to the general population (231–233). Intriguingly, sCD163 secretion by PBMCs *ex vivo* may serve as a biomarker of SSc progression, as increased production of sCD163 by PBMCs was associated with worse prognosis of SSc (233). In addition, urinary sCD163 concentrations were also higher in SSc patients, but the difference was not statistically significant (232). In line with these findings, several reports have shown elevated serum levels of M2-inducing cytokines, i.e., IL-4, IL-13, and IL-10, in patients with SSc (234–236).

A growing body of data has suggested that M2 Mϕ play crucial roles in the activation of resident fibroblasts and the progression of fibrosis, mainly through the release of TGF-β, vascular endothelial growth factor (VEGF), and platelet-derived growth factor (PDGF) (237, 238). Indeed, high levels of TGF-β and PDGF as well as their contribution to SSc have been reported by several groups (46–48). Data from skin samples of SSc patients and healthy control subjects showed that in SSc, the production of TGF-β by Mϕ was partly induced by Cadherin11, which has been implicated in both pulmonary and skin fibrosis (48). In murine studies, blockade of Cadherin11 led to fewer myofibroblasts and decreased dermal thickness in established fibrosis (48). However, whether this treatment may be therapeutically effective needs further verification.

Intriguingly, a recent study revealed that a considerable number of M2 Mo (CD204^+^CD163^+^CD206^+^) co-expressed M1 markers (CD80 and CD86) in the PBMCs of SSc patients, and this subset of cells constituted a significant feature that characterized SSc (23). In addition, down-regulation of the IL-6/signal transducer and activator of transcription 3 (STAT3) signaling pathway was identified in SSc Mo-derived Mϕ (239). These data suggest a more complex activation profile of SSc Mo/Mϕ, consistent with the remarkable plasticity of these cells. Further investigation into the polarization state of Mo/Mϕ in different stages of SSc is needed, and the exact role of these cells should be clarified.

### RA

Mϕ infiltration in the synovia is one of the most important hallmarks of RA. There is ample evidence that the frequency and absolute number of Mϕ are markedly increased in the synovial tissues of patients with RA (4, 5). More importantly, this phenomenon could serve as a reliable biomarker for disease activity. Mulherin et al. showed that synovial Mϕ number correlated positively with articular destruction in RA (240). In a study based on 66 patients with RA, it was found that local disease activity in particular was positively associated with the number of synovial Mϕ as well as levels of IL-6 and TNF-α, two major Mϕ-derived cytokines (241). Accordingly, it was suggested that synovial Mϕ count may also reflect the therapeutic efficacy of RA. An early study by Ghada et al. found that the number of synovial CD68^+^ Mϕ was significantly reduced 12 weeks after treatment with sodium aurothiomalate (242). A further study investigated synovial tissue biopsies from 88 patients with RA participating in various clinical trials, and the authors found that the number of synovial Mϕ correlated significantly with disease activity score, and that a decrease in this number was positively correlated with clinical improvement of RA, independent of the therapeutic strategies these patients received (243). In line with these findings, it was important to find that sublining Mϕ did not change in response to placebo or ineffective treatment (243, 244). These findings were corroborated by data from rodent models of arthritis. It was recently shown that experimental arthritis was accompanied by enhanced survival of synovial Mϕ and would be markedly improved in genetically modified mice in which Mϕ were more susceptible to apoptosis (131). In this study, Mϕ survival is induced by increased expression levels of nuclear factor of activated T cells 5, the expression of which is stimulated by the inflammatory tissue microenvironment of the arthritic mice. Importantly, experimental arthritis was significantly alleviated after local Mϕ depletion by knee joint clodronate liposome injection (245). Moreover, inhibition of Mϕ differentiation from Mo also ameliorated synovial inflammation in experimental arthritis (246). These findings suggest that Mϕ play a key role in RA pathogenesis.

A growing number of studies have highlighted the central role of Mϕ activation in RA pathogenesis. To be specific, unrestrained pro-inflammatory M1 polarization with incomplete M2 polarization usually leads to more severe joint pathology, and thus Mϕ polarization modulation usually alters the outcome of experimental arthritis. In a collagen II-induced arthritis mouse model, it was found that cyclophilin A, a potent pro-arthritic protein, aggravated the severity of arthritis through the induction of pro-inflammatory M1 Mϕ polarization and cytokine production in the knee joint (247). On the other hand, efficiently repressed M1 polarization or increased anti-inflammatory M2 polarization suppressed synovial inflammation and held promising potential as a targeted therapy for RA. In collagen II-induced murine arthritis and spontaneous arthritis in Hes1-GFP/TNF-transgenic mice, inhibited M1 polarization and simultaneously enhanced M2 polarization of Mϕ significantly reduced the inflammatory response in the knee joints (248, 249). Likewise, collagen-induced arthritis was efficiently ameliorated by the administration of mesenchymal stem cells, which have potent immunomodulatory capabilities (250–252). In addition, IL-10 was able to suppress the observed effects of pro-inflammatory M1 Mϕ in experimental arthritis, partly due to inhibition of the inflammation-associated nuclear factor kappa-light-chain-enhancer of activated B cells (NF-κB) signaling pathway or pro-inflammatory cytokine secretion from Mϕ (253, 254). Data from murine model of RA showed that synovial tissue-resident Mϕ and MDMs play different roles in experimental RA. Misharin et al. found that Ly6C^−^ Mo are recruited into the synovial tissue and differentiate into pro-inflammatory M1 Mϕ during the effector phase of arthritis, thus driving initiation and progression of joint inflammation. During the resolution phase, these cells are polarized toward an alternatively activated phenotype and contribute to the resolution of arthritis (13). In comparison, synovial tissue-resident Mϕ are anti-inflammatory throughout the course of arthritis and inhibit joint inflammation during the initiation phase (13).

Activated Mϕ are a potent source of various pro-inflammatory cytokines, which are essential mediators of the effects of Mϕ during the development of RA (56, 132, 255). TNF-α is a key cytokine that is produced by synovial Mϕ and is of critical importance in the pathogenesis of RA (51, 249, 256). This cytokine is present in most arthritis biopsies, and its overexpression induces spontaneous inflammatory arthritis, whereas its inhibition suppresses various rodent arthritis models (134, 135). Accordingly, therapeutic targeting of TNF-α signaling has yielded clinical efficacy in patients with established RA, which has also been corroborated by a number of mouse model-based results (257–259). Other Mϕ-derived cytokines such as IL-1, IL-6, and IL-12 are also abundantly present in the arthritic synovium of patients with RA (134, 135). Similarly, they are indispensable for the inflammatory responses in the synovia of patients with RA, and blockade of their signaling pathways improves clinical or experimental arthritis (52–55).

### Multiple Sclerosis (MS)

In progressive MS, central nervous system (CNS) inflammation is characterized by widespread activation of mononuclear phagocytes (MPs), which include both Mo-derived Mϕ and resident microglia (58). These MPs are found in both gray and white matter lesions, are close to degenerating areas, and are associated with chronic tissue damage (11, 12). In addition, in normal-appearing white matter, MP infiltration is associated with the formation of microglial nodules that lead to disease pathology (260). It has been suggested that staging of MS lesions can be determined based on the presence of CD68-positive Mϕ and human leukocyte antigens, together with the degree of myelin loss (59). The detrimental role of MP-driven pathology in MS is also supported by evidence from murine models, which has shown that the overall burden of MPs correlates with brain atrophy (261), impaired neuronal function (262), and decreased regenerative responses (263). These findings are underpinned by evidence from clinical trials, as induction of Mϕ apoptosis by IFN-β showed a significant benefit in MS (264). In addition, in murine models, Mϕ depletion showed significantly suppressed CNS damage and clinical signs of experimental autoimmune encephalomyelitis (265, 266).

Using brain autopsy tissue from patients with MS, Tobias et al. found that the main functional changes in Mϕ and microglia are increased expression levels of molecules associated with inflammation, including CD68 (phagocytosis), human leukocyte antigen (HLA) and CD86 (antigen presentation and co-stimulation), and inducible nitric oxide synthase (iNOS) (microglia activation) (60). Another group, George et al. found that Mϕ of MS patients display deficient SHP-1 mRNA and protein expression, leading to heightened activation of STAT1, STAT6, and NF-κB signaling and a corresponding enhanced inflammatory profile (142). In addition, data from experimental autoimmune encephalomyelitis (EAE), an animal model of MS, has shown a critical role for Mϕ in triggering adaptive immune responses. For example, Mϕ NLPR3 inflammasome plays a key role in inducing migration of autoreactive T cells into the CNS in EAE (144). Mϕ also produce several key cytokines (i.e., IL-1β, IL-6, and IL-23) to promote the generation and maintenance of Th17 cells, a key cell subset mediating CNS autoimmunity in EAE (145–147). In addition, TLR7-mediated productions of IL-6 and B cell-activating factor (BAFF) are crucial cytokines for autoreactive B cell survival and differentiation (150). In consistent with these findings, Mϕ depletion or anti-GM-CSF treatment inhibits the induction of myelin antigen-specific Th17 cells and protects mice from clinical symptoms of EAE (146, 267–269).

Ample evidence indicates that inflammatory Mϕ in MS show abnormal metabolic changes. Generally, Mϕ activated by inflammatory stimuli switch their core metabolism from oxidative phosphorylation (OXPHOS) to glycolysis (61). Recent evidence shows that inflammatory Mϕ accumulate succinate, which inhibits the function of prolyl hydroxylase enzymes during this metabolic shift, thereby inducing the transcription and secretion of IL-1β as an additional pro-inflammatory signal (61). In line with this finding, Luca et al. recently showed that inhibition of succinate release from MPs can reprogram their metabolism back to OXPHOS, resulting in an anti-inflammatory phenotype of Mϕ and ameliorated experimental autoimmune encephalomyelitis (270).

Many lines of evidence indicate that Mϕ play divergent roles in the pathogenesis of MS as they exacerbate tissue injury but also show remarkable growth-promoting and neuroprotective effects (271, 272). Obviously, this dual role of Mϕ in MS can be explained by their polarization state. In fact, both M1 and M2 subsets are present in MS lesions. The pro-inflammatory M1 response is rapidly induced and then maintained at sites of CNS injury. In comparison, the immunoregulatory M2 response is comparatively weaker and more transient (271). Thus, when inflammatory signals released by type 1 MPs are suppressed by neural stem cell-derived immunoregulatory factors, significantly ameliorated CNS inflammation can be observed (270). On the contrary, sodium chloride treatment of Mϕ induced an enhanced pro-inflammatory activity of these cells and aggravated CNS autoimmunity in EAE-diseased mice (273). In addition, IL-33 treatment induced significantly ameliorated EAE, accompanied by M2 polarization of Mϕ. Of note, adoptive transfer of IL-33-treated Mϕ attenuated EAE development, suggesting the importance of IL-33-mediated Mϕ polarization in the development of EAE (274). In consistent with this finding, Miron et al. found that immunomodulatory M2 Mϕ were essential for oligodendrocyte differentiation through activin A production (275). Notably, the dichotomy of Mϕ polarization in MS is not accurate, as the majority of Mϕ in active MS lesions show an intermediate activation status, characterized by the co-expression of both M1- and M2-specific markers (24). In addition to their polarization state, the dual role of Mϕ in MS pathogenesis can also be accounted by the origins of CNS Mϕ. In fact, resident microglia and Mo can both give rise to Mϕ that exhibit distinct expression profiling in the CNS (276). Yamasaki et al. found the distinct functional capacities of these two Mϕ in EAE. They showed that resident macroglia were associated with debris clearance and demonstrated a signature of globally suppressed cellular metabolism during disease initiation, whereas Mo-derived Mϕ were highly phagocytic and inflammatory and actively participated in demyelination demyelination initiation (277).

### Type 1 Diabetes (T1D)

There are scant data describing correlations between Mo and Mϕ counts and T1D development. In one study, the absolute count of circulating Mo was significantly increased in patients with T1D, while the number of CD16^+^ Mo decreased in patients with diabetic complications (62). Unfortunately, this study did not analyze the correlation between Mo number and T1D development. Another study found that decreased Mo counts significantly correlated with insulin resistance in T1D, although this study lacked data on healthy controls and thus could not prove a relationship between Mo number and T1D development (278).

Two independent studies showed that Mϕ from diabetes-prone non-obese diabetic (NOD) mice showed markedly compromised phagocytosis relative to those from normal mice (63, 64). Since Mϕ engulfment of apoptotic cells is an important mechanism of self-antigen clearance, it was thus suggested that deficiencies in apoptotic cell clearance by Mϕ represent a potential factor in predisposition to T1D. In addition, Mϕ from NOD mice were shown to be abnormally activated and exhibited direct cytolytic activity toward islet β-cells (65). Accordingly, *in vivo* depletion of Mϕ by clodronate liposomes abolished diabetes effectively.

In T1D, Mϕ play a key role in triggering the adaptive immune responses. Vomund et al. showed that islet beta cells can transfer some of their secretory granules to resident Mϕ. In autoimmune diabetes, these Mϕ present the transferred antigens to autoreactive CD4^+^ T cells, resulting in the activation of these cells and initiating the autoimmune diabetic process (279). Mϕ are also involved in the trafficking of autoreactive CD8^+^ T cells into the islets. Marro and colleagues found that depletion of Mϕ or genetic ablation of *ifnar* on Mϕ aborted lymphocytic choriomeningitis infection-induced T1D (280). Mechanistically, disrupted type-I IFN signaling in Mϕ restricted trafficking of CD8^+^ T cells into the islets, thus prohibiting the further development of murine T1D (280).

In T1D, the abnormal activation of Mϕ is exemplified by the pro-inflammatory M1 phenotype of these cells, which play a critical role in T1D pathogenesis. The pro-inflammatory serum milieu of T1D patients that favors M1 Mϕ polarization is exemplified by excessive amounts of C-reactive protein (66), IFN-γ (67), CXCL10 (68), and CCL2 (68). This M1 dominance of T1D Mo is reflected in the elevated IL-6- and IL-1β-secreting ability of these cells, regardless of whether they were in a resting state or after lipopolysaccharide stimulation (66, 69). It was suggested that a main function of these two cytokines is to induce the generation of Th17 cells, which is another key cell population in T1D pathogenesis (69). In addition to the aforementioned two cytokines, several lines of evidence have shown elevated levels of Mϕ-derived TNF-α in T1D patients (70, 71). However, the function of TNF-α in T1D pathogenesis seems controversial. Although TNF-α blockade therapy showed clinical efficacy in some cases, others showed disturbance of glycemic control after treatment, and one study even reported induction of T1D during anti-TNF-α therapy in a RA patient (281, 282).

While pro-inflammatory M1 Mϕ promote T1D development, adoptive transfer of immunosuppressive M2 Mϕ reduces the onset of T1D in NOD mice (283). In fact, more than 80% of NOD mice were protected against T1D for at least 3 months after a single transfer of M2 Mϕ, even if the treatment was conducted just prior to clinical onset. Moreover, *in vitro* induced M2 Mϕ can also reduce hyperglycemia, kidney injury, and insulitis in diabetic mice (284).

The pancreas contains both MDMs and resident Mϕ that exert different functional capacities. Bone marrow Mϕ have been found to prevent stem cell mobilization into peripheral blood in diabetic mice (285). In contrast, the islet resident Mϕ exhibit an activation signature with higher expression of various pro-inflammatory cytokines and mount an inflammatory immune response in NOD mice (286). Consistent with these findings, in a study conducted in C57BL/6 mice, islet Mϕ express genes and cell surface markers that categorize them as M1-like and exhibited typically pro-inflammatory characteristics. In contrast, the interacinar Mϕ expressed M2-like transcripts and exhibited anti-inflammatory and tissue-supportive functions (287). Accordingly, depletion of islet resident Mϕ through CSF-1 neutralization resulted in reduced CD4^+^ T cell infiltration in the pancreatic islets, impaired presentation of insulin epitopes to T cells and reduced severity of autoimmune diabetes (288).

### PBC

In 1994, Mathew et al. found that while Kupffer cell counts were not altered significantly in stage 1 and 2 PBC, increased Kupffer cell numbers were clearly identified in periportal and periseptal zones of stage 3 PBC and in the parenchymal areas of stage 3 and stage 4 cases (10). This finding was supported by another independent study (72). In contrast to these findings, the work of Leicester and colleagues showed that the total number of CD68^+^ Mϕ in the liver remained constant at different stages of fibrosis and did not differ significantly from that of controls (73). This discrepancy may result from distinct disease staging strategies or different hepatic Mϕ immune-staining and quantification methods. In addition to Mϕ, several lines of evidence also showed increased Mo counts in PBC patients. Leicester et al. revealed that while few CD14^+^ Mo could be observed in control livers, these cells were increased markedly in PBC livers, especially in patients with advanced stage of fibrosis (73). The work of Peng et al. showed that the frequencies of peripheral blood CD14^high^CD16^+^ and CD14^low^CD16^+^ subpopulations of Mo were elevated in patients with PBC (74). Intriguingly, the frequency of CD14^low^CD16^+^ cells was positively associated with disease progress. Consistent with these findings, increased levels of Mo chemotactic proteins were also identified in PBC livers (289). These findings are supported by data from murine models of PBC. In dominant-negative TGF-β receptor type II transgenic mice, clusters of Mϕ are observed in the parenchyma and portal tracts of the liver (290). In another PBC mouse model, the 2-octynoic acid-conjugated bovine serum albumin immunization-induced autoimmune cholangitis, interestingly, it was found that while MDMs (CD11b^hi^F4/80^int^CX3CR1^hi^) were enriched around the portal triads, liver resident Kupffer cells (CD11b^int^F4/80^hi^CX3CR1^neg^) were significantly reduced (161). In this study, MDMs play a key role in the development of experimental PBC, as inhibition of their recruitment either by genetic deletion of CCR2 or by pharmacological antagonization of CCR2 resulted in ameliorated autoimmune cholangitis (161).

The dysfunction of Mϕ in PBC is reflected in several findings. In 2005, Mao et al. showed that Mo isolated from the peripheral blood of patients with PBC were more sensitive to toll-like receptor (TLR) ligation and thus produced higher levels of pro-inflammatory cytokines (75). This finding was supported by another independent study, which demonstrated that the expression of TLR4 and its negative regulator RP105 were altered on PBC Mo, making them hyperreactive to LPS and leading to increased production of various pro-inflammatory cytokines (78). In an *in vitro* co-culture model using human peripheral blood Mo and T cells, it was shown that circulating CD14^low^CD16^+^ Mo could promote Th1 cell proliferation by IL-12 production and direct contact of CD4^+^ T cells (presumably through HLA-DR-, CD80-, and CD86-mediated mechanisms). In line with these findings, circulating CD14^low^CD16^+^ Mo were positively associated with Th1 cell frequency in PBC patients (74). Other molecules, such as Siglec-1, were also found to be abnormally overexpressed by PBC Mo (76). A great breakthrough in the abnormally altered functions of Mo and Mϕ in PBC may be achieved in studies illustrating their ability to recognize anti-mitochondrial antibody (AMA)-apotope complexes (77, 164). Apoptotic biliary epithelial cell-derived autoantigens might remain immunologically intact and can be recognized by circulating AMAs in apoptotic bodies (164). Of note, these AMA-apotope complexes are capable of activating Mo-derived Mϕ of the liver, thus stimulating the secretion of various pro-inflammatory cytokines from these cells. This effect leads to further biliary epithelial cell apoptosis, thus perpetuating local inflammation and eventually causing bile duct damage (77).

Many lines of evidence indicate a pro-inflammatory M1 polarization of Mϕ in PBC. These Mϕ express high levels of TLR4 and are highly sensitive to endotoxin stimulation, leading to markedly increased secretion of several pro-inflammatory cytokines, such as IL-1β, IL-6, IL-8, IL-12, and TNF-α (75, 78). Interestingly, endotoxin, which is a strong stimulator of M1 Mϕ activation, is increased in biliary epithelial cells of patients with PBC (79). In addition, levels of CD40L, which interacts with its corresponding receptor CD40 and mediates potent inflammatory signals, are significantly elevated in PBC Mϕ (72). The same study also found that this increase in CD40L expression was mainly stimulated by LPS and IFN-mediated signals.

### SS

Increased levels of peripheral mature (CD14^low^CD16^+^) Mo were described in patients with SS (15), even though their direct aetiopathogenic role remains undefined. Another Mo subset, pro-inflammatory CD14^bright^CD16^+^ Mo, is also increased in the salivary glands of SS patients, accompanied by overexpression of IL-34, a cytokine that specifically stimulates the growth and differentiation of Mo (80). In addition, the salivary profile of CCL2, a potent Mo chemoattractant, is highly expressed in patients with SS (85). Until now, there has been no direct evidence concerning the association of Mϕ or Mo numbers with human SS disease activity, even though elevated expression of Mϕ-derived molecules (i.e., molecules of the chitinase family) indeed corresponded to more severe SS (291). In addition, a study analyzing saliva proteomics showed that proteins associated with Mϕ differentiation represented one of the biomarker signatures of SS (292). In mouse models, it has been shown that Mϕ are critical mediators of SS pathogenesis and have intimate crosstalks with autoreactive T cells. Using autoimmune regulator-deficient mice as an animal model of SS, Zhou et al. demonstrated that Mϕ infiltration the limbus, corneal stroma, and lacrimal glands were mediated by autoreactive CD4^+^ T cells (293). Importantly, local infiltration of Mϕ correlates with ocular surface damage, and Mϕ depletion by clodronate liposomes led to significant improvements in lacrimal gland pathology (293), indicating the immunopathologic involvement of these cells in SS. In another mouse model of SS wherein NFS/sld mice are thymectomized on day 3 after birth, Ushio and colleagues found that tissue resident Mϕ of the salivary gland mediated CD4^+^ T cell recruitment by effective production of CCL22 (171). Moreover, CCL22 was found to enhance IFN-γ production from T cells in these mice (171). Of note, numerous CCL22-producing Mϕ can be observed in the salivary gland tissue specimens of SS patients (171).

Functional abnormalities of SS Mϕ are exemplified by impaired phagocytosis ability of them. Mϕ isolated from an SS mouse model showed defective phagocytosis of apoptotic cells (294). This finding is in line with previous reports in SS patients, as Mo from these patients showed reduced engulfment of apoptotic epithelial cells and were unable to promote an immunosuppressant cytokine profile (81). In addition, elevated levels of MIF have been shown to be associated with hypergammaglobulinemia in patients with SS (295).

There is a paucity of data on the polarization of Mϕ in patients with SS. Although Baban et al. reported the presence of M1 and M2 Mϕ along with T and B cells in the salivary glands of SS mouse model, the balance of M1 and M2 Mϕ has not been characterized (296). However, accumulating data indicate that pro-inflammatory M1 polarization is the predominant phenotype of SS Mϕ. It has been reported that systemic and local concentrations of IL-6 are significantly increased in SS patients (82). In addition, serum IL-12 levels are associated with more active disease, while an immunosuppressant cytokine, IL-35, is associated with lower disease activity (83). It has also been shown that peripheral IFN-γ levels are increased in patients with SS (84), which is suggested to be stimulated by the synergistic functions of IL-33, IL-12, and IL-23 (297). Additionally, salivary levels of the pro-inflammatory cytokines and chemokines TNF-α, IL-1β, IL-18, CXCL8, and CXCL10 are also significantly higher in SS patients than in non-SS controls (80, 85–87). Notably, levels of pro-inflammatory cytokines or chemokines that are directly secreted by Mo and Mϕ, i.e., IL-6, IL-18, type I IFN and BAFF, are significantly higher in SS patients (87, 172, 173). In accordance with the increased pro-inflammatory cytokine levels of SS Mo, these cells express reduced levels of NF-κB inhibitor (IκBα), indicating the abnormal activation of the NFκB signaling pathway (88). In addition, Adrienne et al. used freshly isolated peripheral blood Mo and found that SS-associated microRNAs collectively suppressed immunoregulatory TGF-β signaling as opposed to the pro-inflammatory IL-12 and NF-κB signaling pathways (170). Interestingly, in thymectomized NFS/sld mice, an animal model of SS, tissue resident Mϕ of the salivary gland contain two main subsets (CD11b^low^F4/80^+^ and CD11b^high^F4/80^+^) (171). These two subsets of Mϕ display different phenotypes and functions. For example, CD11b^low^F4/80^+^ Mϕ express higher levels of pro-inflammatory M1 markers including MHC-II, CD11c, and CD86, while CD11b^high^F4/80^+^ Mϕ express higher levels of M2 markers such as CD206 and CD204 (171). In addition, CD11b^high^F4/80^+^ Mϕ showed significantly higher phagocytic activity compared with CD11b^low^F4/80^+^ ones (171).

### Celiac Disease

Numerous CD68^+^ tissue Mϕ were present in duodenal biopsies from patients with celiac disease (7). Of note, these Mϕ showed strikingly impaired phagocytosis ability, as reduced expression levels of Mϕ-associated scavenger receptors, i.e., CD36, thrombospondin-1 and CD61, were identified in the duodenal mucosae of patients with the active phase of celiac disease, accompanied by the accumulation of apoptotic bodies in these areas (89). However, direct evidence for the phagocytosis ability of Mϕ is lacking. In addition, Mϕ from patients with celiac disease exhibit greater antigen-presenting ability, which is exemplified by the upregulated expression of the co-stimulatory molecules CD80, CD86, and CD40, in concert with higher CD40L expression and a more highly activated state of T cells (90, 91). However, more direct evidence is warranted to support this conclusion.

The cytokine milieu of patients with celiac disease implicates a simultaneous M1- and M2-related profiles. For one thing, significantly higher levels of M1-associated pro-inflammatory cytokines, i.e., IFN-γ, IL-1β, TNF-α, and IL-8 have been identified in celiac disease sera (22). More specifically, gliadin peptides could induce significantly higher levels of IL-8 and TNF-α production by Mo from patients with celiac disease relative to those from healthy donors. This pro-inflammatory cytokine secretion is accompanied by a more pro-inflammatory activation state of Mo expressing higher levels of M1 markers, i.e., CD80, CD86, and CD40, as well as higher activation of the NF-κB signaling (90). In addition, it was shown that gliadin fragments could induce RAW264.7 cells and mouse peritoneal Mϕ to secrete TNF-α and CCL5, and to produce increased levels of nitric oxide in the presence of IFN-γ, which is also associated with the activation of NF-κB signaling (298–300). The interaction of gliadin with Mϕ involved a myeloid differentiation factor 88 (MyD88)-dependent pro-inflammatory cascade, while this was neither TLR2- nor TLR4-dependent (176). Intriguingly, even in patients with celiac disease on a gluten-free diet whose duodenal biopsy specimens are histologically normal, intraepithelial lymphocytes and intestinal epithelial cells exhibit increased expression of TNF-α and MIF (301). This may help explain the rapidity with which the celiac mucosa responds to gliadin challenge.

Additionally, M2-associated immunosuppressive cytokines are also frequently detected in celiac disease. For example, IL-10 concentration is significantly higher in celiac disease sera (22). Importantly, serum levels of IL-10 is significantly correlated with levels of autoantibody titers (22). In addition, IL-10 polymorphisms are correlated with more severe mucosal damage and early-onset of celiac disease (302), even though IL-10 secretion abnormalities are suggested to be more a cause than a consequence of this disease (303). Using Mo from patients with celiac disease or healthy subjects, Amelia et al. found that gluten peptides induced the expression of arginase 1 and arginase 2, both of which are typical markers of M2 Mϕ (92). This finding was supported by data from the same group showing that gliadin stimulation significantly activated the arginase pathway in human Mo as well as in RAW264.7 cells (93).

### IBD

In IBD, the intestinal mucosa is characterized by extensive Mϕ infiltration (8, 9). Elevated CD68^+^ Mϕ count in the colonic and ileal mucosae were observed in both Crohn's disease (CD) and ulcerative colitis (UC), while a CD163-positive subset in the colon mucosa was increased only in CD but not UC patients (16). In patients with CD, the mesenteric fat tissue also exhibits considerable Mϕ infiltration (9, 304). Regarding circulating Mo, it was found that Mo with a CD14^+^CD16^+^ phenotype are increased significantly and are the main contributor to the inflammatory infiltrate in the CD mucosa, while classical Mo (CD14^hi^CD16^−^) are decreased (94, 95). A dramatic increase in peripheral CD14^+^CD16^+^ Mo was observed in patients with active CD, particularly in those with colonic involvement and a high Disease Activity Index (95). Intriguingly, a significant correlation between the percentage of CD14^+^CD16^+^ Mo and clinical activity index has been shown in both CD and UC patients, suggesting the potential involvement of this cell subset in the inflammatory drive of IBD (305). Of note, computational simulations conducted by Wendelsdorf et al. identified that Mϕ and their mechanisms of plasticity are key reasons for mucosal inflammation (188).

The expression level of aldehyde dehydrogenase (ALDH), which is necessary for the synthesis of retinoic acid, is significantly reduced in Mϕ populations of the UC colon, both in active disease and remission (8). Given that retinoic acid has important immunoregulatory properties and is critical for the generation of regulatory T cells (Tregs), local suppressive failure due to a lack of retinoic acid may be involved in driving UC. In line with this finding, Treg numbers in UC patients were lower than that of healthy controls, and Treg number was negatively associated with the clinical activity index of UC (306). In comparison, the percent change in ALDH^+^ Mϕ in CD is controversial, as one study showed that this fraction is similar to that in controls, while another study identified up-regulated ALDH activity in CD14^+^ Mϕ from CD patients (8, 307). CD Mϕ also showed an abnormally accelerated breakdown of pro-inflammatory cytokines due to faster lysosomal degradation, while cytokine messenger RNA showed normal stability and levels (96). This was shown to lead to impaired neutrophil attraction, causing defective bacterial clearance and thereby boosting the formation of granulomas. However, this case differs strikingly from UC Mϕ, which showed similar or even significantly higher secretion of various cytokines relative to healthy controls in the same study. In addition, there is proof that IBD patients showed defective Mo GM-CSF receptor (CD116) expression and function, which was more prominent in UC than in CD patients, indicating a causal link between the innate immune defect in IBD patients and Mo CD116 expression (97). Intriguingly, CD116 expression in IBD patients was independent of current medications and was not influenced by disease activity.

Several studies have reported the potential interactions between colonic Mϕ and lymphocytes in IBD. Abnormally activated intestinal Mϕ in CD patients produce various cytokines (i.e., IL-1β, IL-6, IL-23, TNF-α, and TNF-like protein 1A) necessary for T cell differentiation, specifically promoting the generation of Th1 and Th17 cells (191–194). A subset of CD14 and CD209 dual positive Mϕ in the lamina propria also possess potent antigen-presenting ability and can strongly evoke the differentiation of Th1 and Th17 cells (194). In addition, these Mϕ can induce the proliferation of naive CD4^+^ T cells (194). Similarly, in UC patients, IL-23 from CD68^+^ Mϕ promotes the differentiation of Th17 cells, which are important contributors to the pathogenesis of UC (195–197). In addition, Mϕ-derived IL-23 can strongly promote the activation and cytolytic activities of intestinal NK cells crucially contributing to tissue pathology of UC patients (195). Data from murine model-based studies showed that adoptive transfer of M2a Mϕ to IBD mice increased Th17 and Treg generation, while M1 Mϕ contributed to the disruption of the intestinal epithelial barrier during IBD development (308, 309).

The polarization profile of IBD Mϕ is a complex issue. In CD, Mϕ are more polarized to an M2 profile, which is reflected by several findings. First, CD163 is expressed on a substantial percent of Mϕ in the colonic mucosa as well as in the peripheral blood of CD patients (16). In addition, sCD163 levels are significantly increased in CD patients (310). Upon successful treatment, serum sCD163 levels are dramatically decreased (310). Second, large numbers of Mϕ are found in fibrotic lesions of CD patients, consistent with the potent tissue-repairing and pro-fibrotic capacity of M2 Mϕ (311, 312). Third, defective bacterial clearance by Mϕ is frequently observed in CD patients, which is presumably due to the impaired pro-inflammatory cytokine secretion of these cells (96). Fourth, IL-13, which is a potent M2 Mϕ inducer, was dramatically upregulated in CD patients (100). In comparison, the Mϕ polarization profile seems much more complex in UC patients. The fact that CD163^+^ Mϕ numbers and serum sCD163 levels are increased in UC patients, coupled with the finding that CD206^+^ Mϕ are enriched in the injured mucosa of these patients, indicates an M2 polarization profile for these Mϕ (16, 101). However, the continuous excessive inflammation in the gut mucosa of UC patients, as well as the significant increase in pro-inflammatory M1 while decrease in M2 Mϕ accompanied by suppressed IL-10 production in mouse models of UC also points to the evident M1 polarization of these Mϕ (98, 99). In various mouse models of IBD, inhibition of the pro-inflammatory activities of M1 Mϕ or induction of tissue-repairing/immunomodulatory M2 Mϕ usually results in attenuated experimental IBD (185, 187, 313, 314).

## Conclusions and Future Perspectives

**Figure 1:**
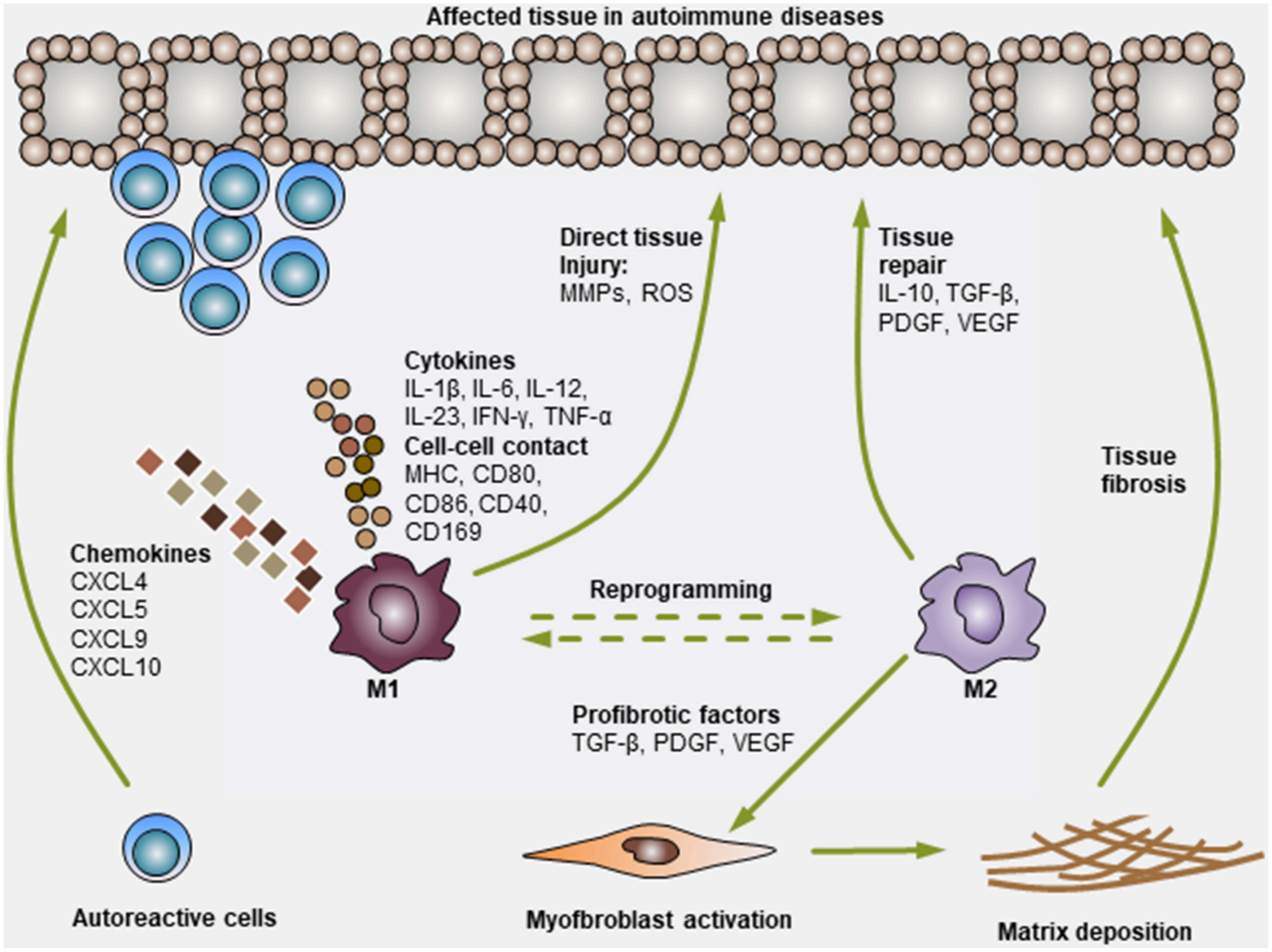
Modulation of autoimmune diseases by Mo and Mϕ. Mo and Mϕ are key players in autoimmune diseases. During the development of autoimmune diseases, pro-inflammatory M1 Mo or Mϕ can secrete various chemokines to recruit additional immune cells (i.e., T cells, B cells, neutrophils, NK cells, and NKT cells) to the affected tissues. Then, Mo or Mϕ can activate these cells via the secretion of various pro-inflammatory cytokines (i.e., IL-1β, IL-6, IL-12, IL-23, IFN-γ, and TNF-α) or through direct cell-cell contact (antigen presentation: MHC, co-stimulation: CD80, CD86 and CD40, and adhesion molecules: CD169). In addition, Mo or Mϕ can also exert direct tissue injury functions by producing matrix metalloproteinases (MMPs) and reactive oxygen species (ROS). Consequently, the activation of Mo or Mϕ and other immune cells synergistically leads to tissue damage. On the other hand, M2 Mo or Mϕ mediate immunosuppressive or tissue-repairing effects during this process, mainly by producing cytokines (i.e., IL-10 and TGF-β) and growth factors (i.e., PDGF and VEGF). M2 Mo or Mϕ can also secrete various pro-fibrotic factors, such as TGF-β, PDGF and VEGF, to activate myofibroblasts in certain tissues, leading to extracellular matrix deposition and fibrosis generation (i.e., cases in PBC and SSc).

In the present review, we mainly discussed the association of Mo/Mϕ with the development of certain autoimmune diseases. It has been quite well elucidated that Mo/Mϕ are key component of the innate immune system and are involved in both amplifying and suppressing inflammation (2). Mounting evidence suggests that these cells participate in the pathogenesis of autoimmune diseases, mainly through their remarkably pro-inflammatory or fibrogenic properties (1, 2). As discussed above, in different autoimmune diseases, the heterogeneity of Mo/Mϕ subpopulations varies dramatically, and their polarization profile usually plays a key role in disease progression (Figure 1). However, in many autoimmune diseases, the phenotypic and functional characteristics of Mo/Mϕ have not been classified unambiguously, as many pro-inflammatory M1-polarized Mo/Mϕ simultaneously express M2-related markers or exhibit immunomodulatory functions (19–22). In addition, in several cases, Mϕ activation is a dynamic and reversible event in which pro-inflammatory Mϕ can be re-programmed into Mϕ with immunosuppressive or tissue-repairing cells by local microenvironment (13, 25). Thus, future investigation into explaining the seemingly opposing phenotypic and functional programs of Mo/Mϕ and identifying the dynamic changes is clearly needed.

Several possible mechanisms responsible for Mϕ phenotype in autoimmune diseases in general have been suggested by recent findings. For example, genome-wide association studies have identified several candidate genes responsible for the pathogenesis of autoimmune diseases. Among the susceptibility genes, *HLA*, which is closely with the antigen-presenting ability of Mϕ, has been suggested to be involved in the development of SLE (315), SSc (316), RA (317), MS (318), T1D (319–322), SS (323), Celiac disease (324), and IBD (325). In addition, protein tyrosine phosphatase, non-receptor type 22 (*PTPN22*), which can be expressed in Mϕ and controls Mϕ activation and polarization, has been identified as a risk gene for RA (317) and IBD (17). Interferon regulatory factor 5 (IRF5), which is mainly expressed by myeloid cells and is a key regulator of Mϕ activation and polarization, has been identified as an important predisposed factor in patients with SLE (326), SS (323), RA (327), PBC (328), and IBD (329, 330). However, functional studies investigating the actual function of these genes in Mϕ should be done to confirm whether they really play a critical role in controlling Mϕ activation in autoimmune diseases.

In recent years, mounting reports have overturned the long-held knowledge that Mϕ in the adult are merely replenished by circulating Mo from bone marrow progenitors (331–333). The new paradigm supports that some Mϕ are embryo-derived and are maintained by self-renewal independent of hematopoietic contribution (332). Intriguingly, this heterogeneity of Mϕ results in distinct phenotypes and, more importantly, totally different biologic functions (334, 335). Thus, it is necessary for future studies to elucidate the roles of tissue-resident Mϕ and bone marrow-derived Mϕ in the initiation, progression and termination of different autoimmune diseases.

Although Mo and Mϕ play a key role in the pathogenesis of certain autoimmune diseases, the development of these diseases is not solely Mo/Mϕ-dependent, and this process involves the interplay of these cells with other immune cells, i.e., autoreactive T and B cells (2). However, most studies fail to explore the interactions of Mo/Mϕ with other immune cells in the local microenvironment. Thus, future work is needed to better determine the synergistic effects and related mechanisms of the interactions between Mo/Mϕ and other immune cells in the development of autoimmune diseases.

To date, although the functions of Mo/Mϕ in several autoimmune diseases have been determined, the clinical translation of this knowledge is still challenging. Certain Mo- or Mϕ-targeted therapies have been developed (see Table 3), but whether they are more effective and safer than traditional treatment remains to be verified, and some of them have already proven disappointing (52, 54, 281, 282). However, this does not rule out a potential effective role for Mo/Mϕ as an attractive therapeutic strategy for autoimmune diseases. Thus, further studies are needed to elucidate a more detailed and comprehensive mechanism of Mo/Mϕ regulation in autoimmune diseases; such work, coupled with a wider understanding of the determinant factors of autoimmune diseases (i.e., sex, age, genetics, and environmental factors), which act together but differ between patients, will probably lead to the development of more specific and effective therapies in the future.

**Table 3:** Pathogenic functions of Mo and Mϕ in autoimmune diseases and the relevant treatment strategies.

**Diseases**	**Pathogenic functions**	**Relevant strategies of disease treatment**
SLE	Enhanced ability to activate autoreactive T and B cells (28, 336, 337). Higher antigen-presenting ability (29, 30). Impaired clearance of apoptotic cells and immune complexes (34, 35).	Adoptive transfer of M2 Mϕ in mouse model (216). Induction of M2 polarization in patients (217). Blockade of TNF-α (222).
SSc	Contributing to skin fibrosis (44). Mo count correlates with disease activity (42). Potentially mediate genetic susceptibility to SSc (229).	Suppression of M2 Mϕ by tocilizumab (338). Blockade of TGF-β (339).
RA	Mediation of local and systemic inflammation (56, 340). Cartilage degradation (136). Synovial Mϕ count correlates with local disease activity (241).	Blockade of TNF-α (257). Blockade of IL-1 (52). Blockade of IL-6 (54).
MS	Higher antigen-presenting ability (60). Positively associated with disease pathology (260, 264). Mediation of myelin damage through iNOS production (60). Mediation of neurotoxicity (271).	IFN-β-induced Mϕ apoptosis (264). Gc protein-derived Mϕ-activating factor treatment (341). Induction of M2 Mϕ (342).
T1D	Impaired clearance of apoptotic cells (63, 64). Mediates death of islet β-cells (65). Production of reactive oxygen species (343).	TNF-α clearance from the circulation (281). Adoptive transfer of M2 Mϕ in mouse models (283, 284). TGF-β-engineered mesenchymal stem cell treatment in mouse model (344).
PBC	Higher ability to produce pro-inflammatory cytokines (75, 78). Promoting Th1 activation (74). Apoptosis induction of biliary epithelial cells (77, 164). Frequency of CD14^low^CD16^+^ cells correlates with disease progression (74).	Induction of M2 Mϕ by MSC transplantation (345, 346). Blockade of TNF-α (347, 348). Blockade of IL-12/IL-23 (349). Blockade of CCR2/CCL2 signaling (161).
SS	Impaired clearance of apoptotic cells (81). Chitinase levels correlates with SS severity (291). Mediation of local and systemic inflammation (87, 88, 170, 172, 173). MIF concentration correlates with hypergammaglobulinemia (295).	Blockade of TNF-α (ineffective) (350, 351).
Celiac disease	Enhanced ability to activate autoreactive T cells (90, 91).	Parasitic helminth infection (352). TNF-α blockade (353, 354).
IBD	Mediation of local inflammation (94, 355). Percentage of CD14^+^CD16^+^ Mo correlates with disease activity (305). Boost the formation of granulomas in CD (96).	IL-6 blockade (356). IL-12/IL-23 blockade (357). IFN-γ blockade (358). TNF-α blockade (359). MMP9 blockade (360). Allogeneic mesenchymal stem cell transplantation (361, 362).

## Author Contributions

W-TM and D-KC designed the structure of this article. W-TM wrote the manuscript. FG and KG revised the manuscript. All authors have reviewed the final version of this article.

### Conflict of Interest Statement

The authors declare that the research was conducted in the absence of any commercial or financial relationships that could be construed as a potential conflict of interest.
